# Surgery

**Published:** 1892-09

**Authors:** W. J. Penny


### SURGERY.

Cases of trephining the spinal canal, more especially for paraplegia due to Pott's disease or traumatism, have on several occasions during the last few years been brought before the profession.

Taking first the question of Pott's disease, we find that in 1888 Professor Macewen brought forward[1] two cases of paraplegia, due to the pressure of connective tissue tumours at the seat of angular curvature of the spine. The first case was that of a boy aged 9, with complete sensory and motor paraplegia, with incontinence of urine and fæces existing for a period of two years, and which had been absolute for the last eighteen months. The limbs were livid and cold, and affected with spastic rigidity. The laminæ of the 5th, 6th, and 7th dorsal vertebræ were removed; there was no pulsation of the cord at that part. Between the theca and the bone was a fibrous neoplasm $\frac{1}{8}$ inch thick; this was dissected off, and the cord was then able to expand backwards, and shortly began to pulsate. The boy gradually improved; after six months he was able to go about without support, and after several years joined in all the school games, including football. The second case was more aggravated. On operation, the portion of cord exposed was found shrunken to half its normal dimensions, and it lay like an inanimate rod. The recovery, though apparently hopeless, was satisfactory, the patient being able to walk a quarter of a mile and attend to her household duties. A third case was also successful; a fourth and fifth were not so: one died a week after the operation, the other some months later, of general tuberculosis. In both of these latter the temperature was high prior to operation, and Dr. Macewen now considers that this renders operation unadvisable.

[1] *Brit. Med. Journ.*, 1888, vol. ii., p. 308.

About the same time, Mr. Thorburn [1] operated on a patient who was sinking rapidly; operation gave exit to a little pus, with some relief, but the patient died the following day.

In October, 1891, Mr. W. A. Lane, of Guy's Hospital, brought before the Clinical Society of London [2] eleven cases of compression paraplegia due to spinal caries. He found that in every case, except one where the granulation material had not yet broken down, the cord was compressed by an abscess, and that several of these patients would in all probability have died of chest or bladder complications from which they were suffering, if relief had not been afforded by operation; that in only one case, in which the child was extremely feeble, was death due to the surgical interference. The majority were greatly benefited. In only one case was the subsequent formation of tuberculous material so rapid as to obliterate very quickly the benefits derived from two operations. Mr. Lane laid it down, that every case of spinal caries accompanied by paraplegia should be operated on with as little delay as possible. In the discussion which followed Mr. Lane's paper, Mr. Davies Colley described a case he had operated on twice, removing much caseous material and opening an abscess cavity; but the child was distinctly worse after each operation. Mr. Bowlby also described two cases on which he had operated. He found no pus in either; but both rapidly improved after the operation.

Mr. Southam [3] showed a child æt. 6, before the Clinical Society of Manchester, on whom he had operated two years previously for spinal caries, causing complete paralysis of upper and lower extremities, with incontinence of urine and fæces. The spines and laminæ of the fourth cervical to first dorsal vertebræ inclusive had been removed. When shown, the child could walk without assistance, and had regained control of the sphincters. Mr. Southam remarks that complete rest in the recumbent position, with fixation of the spine, will, as a rule, suffice to cause the paralysis in these cases to disappear; and that operative treatment is only indicated in a small proportion of cases, and should not be adopted until palliative treatment has been given a fair trial, and then only when no benefit has resulted, or when the symptoms are progressing in spite of treatment. The pressure is generally due to a localised pachymeningitis.

M. Boiffon [4] operated on a lad of 16 with cervical caries

[1] *Brit. Med. Journ*, 1888, vol. ii., p. 665. [2] *Ibid.*, 1891, vol. ii., p. 949. [3] *Ibid*, Feb. 6, 1892. [4] *Ibid.*, May 7, 1892. Supp.

and paralysis of upper and lower extremities. The spinous processes and laminæ of the third and fourth cervical vertebræ were removed. The patient gradually improved, and four months after the operation could walk.

At the present time, I have two cases under my care at the Bristol General Hospital. One is a strumous lad of 18, with marked angular curvature in the dorsal region. Paralytic symptoms showed themselves last November and, in spite of treatment, developed to complete motor paralysis of legs, with spastic rigidity and wasting, and inability to localise sensations. He also had incontinence of urine. His general health became seriously impaired, with pyrexia, and his urine was ammoniacal and purulent. On consultation with my colleagues, I trephined over the most prominent part of the angle, and pus came welling out beside the trephine. On removing the laminæ (3), a cavity was found in front of the cord the size of a large walnut, and between the laminæ and the cord a mass of thickened tissue. The cavity was scraped and the tissue removed. The patient bore the operation remarkably well, and the wound soon healed. No stitches were introduced. After the operation, there was less spasm, especially of the right leg, and he gradually regained control of the urine, which has become more normal in character. After three months, he has regained power of voluntary movement in his toes, and his general condition is markedly better. The second case is a girl æt. 6, with paraplegia and incontinence of urine, with a marked angular curvature in the dorsal region. She was in a very emaciated condition, with bed sores, when admitted, but has since improved considerably in general condition under tonic treatment and rest, and has found great relief from a Bavarian plaster jacket. Under these circumstances I propose to persevere with the treatment for some time longer.

Turning now to the question of laminectomy for fracture of the spine, we find that at Glasgow, in 1888, Professor Macewen brought forward[1] a case of complete motor paralysis of the legs, and incontinence of urine, due to a fracture of the twelfth dorsal vertebra, the result of a coalpit accident. The case was rapidly running to a fatal termination, with pyrexia, ammoniacal urine, etc. When Professor Macewen operated, the laminæ of the twelfth dorsal vertebra were found fractured and depressed, and between it and the theca was a connective tissue tumour nearly ¼ inch thick, and

[1] *Brit. Med. Journ.*, 1888, vol. ii., p. 309.

extending from the eleventh dorsal to the second lumbar vertebra. This was removed; the man rapidly improved, and after a year was able to walk without assistance.

About the same time a case of fracture of the cervical vertebræ was published [1] by Mr. Keetley. He trephined three hours after the accident, and found the laminæ of the fourth cervical vertebra pressing on the cord, and removed them as well as those of the fifth. The patient, who was apparently relieved by the operation, died three days after, and at the autopsy the spinal cord was found to be completely severed at the part operated on.

In April, 1889, Mr. Allingham brought forward [2] two cases. In one some improvement took place; the other died seven months later of cystitis, bed sores, etc. In both the wound healed readily; and Mr. Allingham notices that the operation did not affect the patients injuriously in the slightest degree. He suggests waiting a week after the injury, to see if the patient improves; if not, to operate.

Mr. Knox [3] successfully trephined the spine for fracture, with complete loss of power and sensation in the legs. The patient made satisfactory but slow progress towards recovery after the operation, and at the end of twelve months could stand and walk a few steps without support.

Mr. Golding Bird [4] reports a case of fracture, separation and displacement of vertebræ, whereby the laminæ of the 11th and 12th dorsal vertebræ pressed on the cord, causing almost entire paralysis. These were removed, and the patient made an excellent recovery. Eighteen days after the operation, when the nurse's back was turned, she sat up in bed. She left the hospital about two months after, wearing a poroplastic felt jacket and walking perfectly well. Mr. Golding Bird remarks that there is apparently more chance of advantageously relieving pressure when the injury is inflicted by direct violence than by indirect. He adds: "Seeing then the doubt that surrounds the precise nature of the fracture, and knowing what an unfavourable future lies before the patient, a surgeon is surely justified in every case in urging upon the patient the propriety of an exploratory operation, in the hope that the condition found on exposure of the cord may be a relievable one."

M. Audry, of Lyons, [5] operated on a man when he was

[1] *Brit. Med. Journ.*, 1888, vol. ii., p. 421.
[2] *Lancet*, 1889, vol. i., p. 790. [3] *Glasg. Med. Journ.* April, 1891.
[4] *Brit. Med. Journ.*, May 23, 1891.
[5] *Brit. Med. Journ.*, Dec. 5, 1891. Supp., p. 179.

semi-comatose; he did not recover, and at the autopsy the cord was found converted into a semi-fluid mass.

M. Vincent, of Algiers, records[1] 8 cases of trephining the spine for gun-shot injuries, with 5 recoveries and 3 deaths. He classifies the lesions as follows: (1) Simple compression of the cord by effused blood, splinters of bone, or fragments of projectile. (2) Contusion or laceration of the cord caused by the bullet. (3) Lodgment of the bullet in the vertebral column, with or without projection into the canal.

Drs. Church and Eisendrath[2] give an account of 8 cases of spinal cord surgery. One, a fracture, died without operation. The remaining seven were operated on, one for tumour of the cord, a sarcoma: five died, including the case of tumour; two were relieved, one of them making an excellent recovery. In this case the canal was filled by extra-dural bloodclots, and by the operation it was found possible to reduce a displacement and keep the parts in position by suturing two spinous processes together with strong silk.

Several cases of fracture of the spine have been recorded in which the patients recovered without operation.

Dr. Beevor[3] showed at the Medical Society of London a man who had fallen 40 feet and injured his back. The vertebræ implicated were from the 10th dorsal to the 2nd lumbar, the 12th dorsal being the most prominent. He was unable to walk, and at first lost control of his sphincters; but this soon passed off, and after ten months he could walk with assistance.

At the Hunterian Society Mr. Tatham showed[4] a man who had fallen in a fit, forcibly flexing his neck. Complete paralysis of sensation and motion below the neck resulted, and respiration and deglutition were difficult; he also had incontinence of urine and fæces. He remained in this state for three months, taking only liquid nourishment, when movement and sensation began to return in the right fingers and toes. After fourteen months he could stand, and when exhibited could walk 10 to 14 miles; there was still some irregularity and thickening about the 3rd cervical vertebra.

Dr. Ryan[5] reports a case of fracture in the dorso-lumbar region, with complete paraplegia. He treated the man with iodide of potassium pushed to iodism, and the actual cautery freely applied to the back, and, as the case showed improve-

[1] *Brit. Med. Journ.*, Dec. 5, 1891. Supp., p. 179.
[2] *Amer. Journ. Med. Sci.*, April, 1892.
[3] *Brit. Med. Journ.*, 1888, vol. ii., p. 994.
[4] *Ibid.*, p. 1163.
[5] *Cincinnati Lancet-Clinic*, June 18, 1892.

ment, a plaster-of-Paris jacket. The man improved so that he could go on crutches.

Two cases of concussion of the spine, with paraplegia, were under the care of Mr. Le Gros Clark.[1] In one case there was retention of urine and also a fracture of the spinous process of the 4th lumbar vertebra, with depression. He gradually regained power, and was discharged in a fortnight. The other, a slighter case, left the hospital in a few days.

A case is reported[2] by Mr. D. L. Davies, in which a fracture of the body and laminæ of the 5th cervical vertebra occurred without displacement; paraplegia ensued and the patient died. At the P. M. E. a considerable quantity of dark blood was found in the spinal canal, being sufficient in amount to cause some compression of the cord.

It is clear from the cases referred to that trephining the spinal canal will shortly become established as a recognised operation in surgery, and one that holds out a fair chance of recovery in what would otherwise be, in many cases, a hopeless condition. The cases of paraplegia, due to Pott's disease, operated on successfully by Prof. Macewen, Messrs. Lane, Bowlby, Southam, and Boiffon, and the one under my care at present, are sufficiently encouraging to induce one to operate in similar bad cases; for such cases if left alone have probably only a fatal termination before them at no distant date. The majority of surgeons appear to agree that the operation itself is not attended with much shock or much risk; *i.e.*, not more than trephining the skull, for instance.

Fears have been entertained that removal of several laminæ and spinous processes would tend very materially to weaken the patient's back; but such does not appear to be the case, as we read of one of Professor Macewen's cases playing football some years after; in his case the spines and laminæ of three dorsal vertebræ were removed. Mr. Golding Bird's case sat up in bed eighteen days after two spines and laminæ had been removed. Probably in these cases, as M. Moty tells us[3] (as the result of a *post-mortem* examination of a patient from whom part of a vertebral arch had been removed), the gap was filled with fibrous tissue.

In these cases of caries the pressure appears to be due to some variety of inflammatory growth, either a connective tissue neoplasm, or granulation tissue, or caseous material, or a distinct abscess, rarely to pressure by bone. When no pus

[1] *Amer. Pract. and News*, June 18, 1892, p. 406.
[2] *Brit. Med. Journ.*, June 25, 1892. [3] *Ibid.*, Dec. 5, 1891. Supp., p. 179.

is found, benefit appears to result simply from removing the laminæ, as in Mr. Bowlby's cases.

We must not forget that many of these cases recover without operation. Professor Erichsen says that the paraplegia will pass off after a few months or a year or two, even though electric irritability and excitability have for a time been completely lost. And the majority of surgeons only recommend operation when cases get worse in spite of treatment. Dr. Ridlon has compiled an interesting report[1] on this subject. The difficult point to decide, as in so many other strumous affections, is when to operate, and when further treatment by rest, tonics, hygiene, &c., is futile by itself. Mr. Lane speaks very decidedly and says that every case of spinal caries accompanied by paraplegia should be operated on with as little delay as possible; but the majority of surgeons advise treatment by tonics, rest, and attention to diet and hygiene first, and only when that has failed to effect improvement, to operate. Operation in this affection, as in many others, is not likely to be of much service when the patient is *in articulo mortis*.

Turning now to the question of when to operate in fracture or traumatisms of the spine, we have again a rather difficult problem to solve. On the one hand there are severe cases such as those described by Dr. Beevor, Mr. Tatham, Dr. Ryan, and Mr. Le Gros Clark, which recover without operation, and on the other, if one leaves a case of compression unrelieved for a time, there is the risk of destructive changes taking place in the cord itself, as Mr. Golding Bird has particularly called attention to. Mr. Allingham suggests waiting a week after the injury, to see if the patient improves; if not, to operate. Dr. Ryan draws a distinction between the different regions of the cord: he would operate earlier in the dorsal region than in the lumbar. In fracture in the latter region, as I had occasion to verify the other day at a P. M. E., the cauda equina becomes splayed out and adapts itself to any crevices that may remain; whereas a similar displacement in the dorsal region, when the cord is a single rod, would cause injurious pressure, and possibly division of it. The experience I have had of operating in two cases, one of fracture in the cervical region, and one in the lower dorsal, is not very encouraging, as in both the cord was completely crushed. In one I removed several considerable-sized fragments of bone which were pressing on the cord. In a case which came under my care a few weeks ago, the man had paraplegia, but not absolute, as he could move his feet a little.

[1] *Epit. Med.*, Feb., 1892.

His general condition was too bad to admit of operation, as he had pneumonia, and had also passed a considerable quantity of blood with his urine. He died a few days after the accident, and at the P. M. E. we found two large cystic kidneys with a mulberry calculus in each, and these were probably the cause of the hemorrhage. The portions of the spine were very movable, and if the case had been fit for operation Dr. Hadra's suggestion[1] of wiring the spinous processes together would probably have been of great value.

W. J. PENNY.

### OPHTHALMOLOGY.

[1] *Brit. Med. Journ.*, Aug. 15, 1891. Supp., p. 50.

